# Successful Subcutaneous Defibrillator Implantation in a Pregnant Patient With Long QT Syndrome

**DOI:** 10.1016/j.jaccas.2020.12.038

**Published:** 2021-03-03

**Authors:** Rahul Myadam, Sanjaya K. Gupta

**Affiliations:** a University of Missouri-Kansas City School of Medicine, Kansas City, Missouri, USA; b Saint Luke’s Mid-America Heart Institute, Kansas City, Missouri, USA

**Keywords:** pregnancy, ventricular tachycardia, x-ray fluoroscopy, DT, defibrillation threshold, ICD, implantable cardioverter-defibrillator, LQTS, long QT syndrome, SCA, sudden cardiac arrest, S-ICD, subcutaneous implantable cardioverter-defibrillator, TV-ICD, transvenous implantable cardioverter-defibrillator

## Abstract

A 26-year-old woman with recurrent unexplained syncope in the postpartum period was diagnosed with long QT syndrome type 2. Traditional implantation of defibrillator using fluoroscopy became contraindicated after she again became pregnant prior to device implantation. A subcutaneous defibrillator was successfully implanted in the second trimester, after a multidisciplinary evaluation. (**Level of Difficulty: Intermediate.**)

## Graphical abstract

**Figure:**
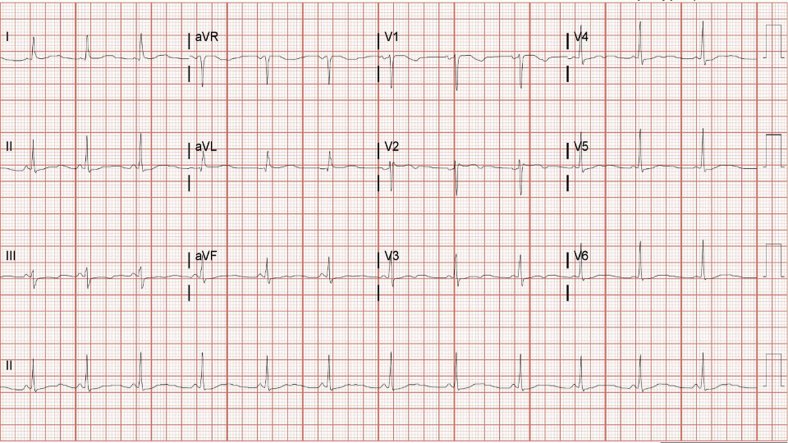

## History of presentation

A 26-year-old woman was referred to the cardiac electrophysiology service to evaluate 3 syncopal episodes over 3 months. Her first event occurred at rest when she had a sudden loss of consciousness associated with urinary incontinence. The second episode happened at night while attending to a crying baby. She had a brief prodrome of light-headedness, followed by loss of consciousness and urinary incontinence. She did not take any medications and had no family history of sudden cardiac arrest (SCA). A complete cardiac and neurological examination did not reveal any abnormalities.

**Learning Objectives**

• To understand the importance of considering congenital LQTS when evaluating syncope in a postpartum woman, and that genetic testing should be ordered after excluding other causes of syncope.
• To describe the indication for ICD therapy in congenital LQTS patients on beta-blockers who survive sudden cardiac death, and that for patients that do not require pacing, a subcutaneous defibrillator is a comparable option to a transvenous defibrillator and is preferred when fluoroscopy is contraindicated.
• To explain the importance of a multidisciplinary team in caring for pregnant patients with high-risk cardiac conditions, such as LQTS.

## Differential Diagnosis

Cardiac arrhythmia, valvular heart disease, neurogenic or vasovagal syncope, or seizures.

## Investigations

**Figure 1:**
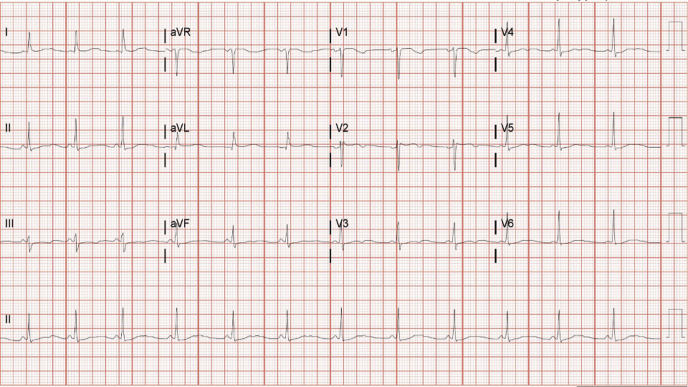
Electrocardiogram Electrocardiogram on presentation after second syncopal episode showing a corrected QT interval of 511 ms.

An initial electrocardiogram showed sinus rhythm with a prolonged QT/QTc interval of 484/511 ms (Figure 1). She had mild hypokalemia, 3.0 mEq/dl (normal 3.5 to 5.2 mEq/dl), that was felt to cause a prolonged QT interval. Neurological workup revealed normal magnetic resonance imaging of the brain and electroencephalogram. After electrolyte replacement, she was discharged with plans for an ambulatory cardiac monitor. However, she had another syncopal episode 3 days later and returned to the hospital. An electrocardiogram showed persistent long QTc interval (486 ms) even though her potassium normalized (3.9 mEq/dl). An echocardiogram demonstrated an ejection fraction of 50% to 55% without valvular abnormalities. Genetic testing for congenital long QT syndrome (LQTS) was ordered due to the typical presentation of syncopal episodes in a postpartum woman with auditory triggers and the absence of other causes of QT prolongation. The genetic analysis showed a mutation in KCNH2, consistent with hereditary LQTS type 2.

## Management

The patient was started on metoprolol tartrate 25 mg twice daily. It was recommended that she undergo implantable cardioverter-defibrillator (ICD) implantation for secondary prevention of polymorphic ventricular tachycardia. However, she unexpectedly had a positive pre-procedural urine pregnancy test. A multidisciplinary team was constituted, including cardiology, anesthesia, and maternal-fetal medicine, given the high risk of SCA during pregnancy and the postpartum period in LQTS type 2. The implantation of the ICD was delayed to the second trimester, given the danger of general anesthesia and narcotics to the fetus in the first trimester (1). She was prescribed a wearable cardiac defibrillator (Life Vest, Zoll Inc., Pittsburgh, Pennsylvania) in the interim, due to the known increased risk of SCA in pregnant LQTS type 2 patients.

At 20 weeks gestation, the patient presented for ICD implantation. She was sedated using propofol and fentanyl and received only a limited amount (15 ml) of lidocaine due to low body weight (45.4 kg) and concern for transplacental transmission. A subcutaneous implantable cardioverter-defibrillator (S-ICD) (EMBLEM S-ICD A209 generator, Boston Scientific, Natick Massachusetts) was implanted via a 2-incision technique, sparing the suprasternal incision (2). The generator was placed between the serratus anterior and latissimus muscles via an intermuscular method. Defibrillation threshold (DT) testing was withheld at the time of implantation. The patient was not exposed to any radiation during the procedure. The peri-procedural fetal monitoring was unremarkable. The patient was discharged post-procedure and took metoprolol throughout her pregnancy.

## Discussion

In patients with congenital LQTS, the measures recommended for the prevention of SCA include beta-blockers, ICD implantation, and left cervicothoracic sympathectomy (3). Rarely, an ICD implantation becomes necessary during pregnancy. The 2017 American Heart Association/American College of Cardiology/Heart Rhythm Society guidelines recommend considering an ICD implantation when necessary in all pregnant women (3). However, novel defibrillator implantation methods need to be considered in pregnancy to prevent fetal radiation exposure. Abello et al. (4) described a pregnant patient with mitral valve prolapse who underwent ICD placement under transesophageal echocardiogram guidance. A few cases of successful device implantation using electroanatomic mapping were reported (5). However, these nonconventional transvenous implantable cardioverter-defibrillator (TV-ICD) implantation methods have limited reproducibility in other settings due to the financial costs and the lack of data on safety outcomes. In this setting, an S-ICD is an alternative with established safety outcomes in nonpregnant women. To date, 1 other reported case of S-ICD implantation in pregnancy was described by Viani et al. (6). Some studies have shown that atrial pacing may be beneficial in preventing polymorphic ventricular tachycardia in LQTS (7). However, in our patient, permanent cardiac pacing was felt to be unnecessary as the arrhythmias were only provoked by pregnancy.

The use of an S-ICD may have an advantage over TV-ICD, especially in younger patients without a pacing indication. A PRospective, rAndomizEd Comparison of subcuTaneOous and tRansvenous ImplANtable Cardioverter Defibrillator Therapy (PRAETORIAN) was a multicenter, prospective, randomized trial that compared S-ICD with TV-ICD and concluded that S-ICD was noninferior to TV-ICD. Lead-related complications occurred more often in TV-ICD (6.6%) versus S-ICD patients (1.4%) (hazard ratio: 0.24; p = 0.001) (8). Moreover, studies have shown that about 20% of transvenous leads fail by 10 years, which increases both the financial costs and the procedural complications associated with TV-ICD (9). Care must be taken when programming an S-ICD to reduce inappropriate therapy.

The role of DT testing during S-ICD implantation is uncertain. Although guidelines recommend routine DT for all patients receiving an S-ICD, this is controversial (10). However, in our patient, DT was withheld at the time of device implantation due to potential risk to the fetus.

## Follow-Up

**Figure 2:**
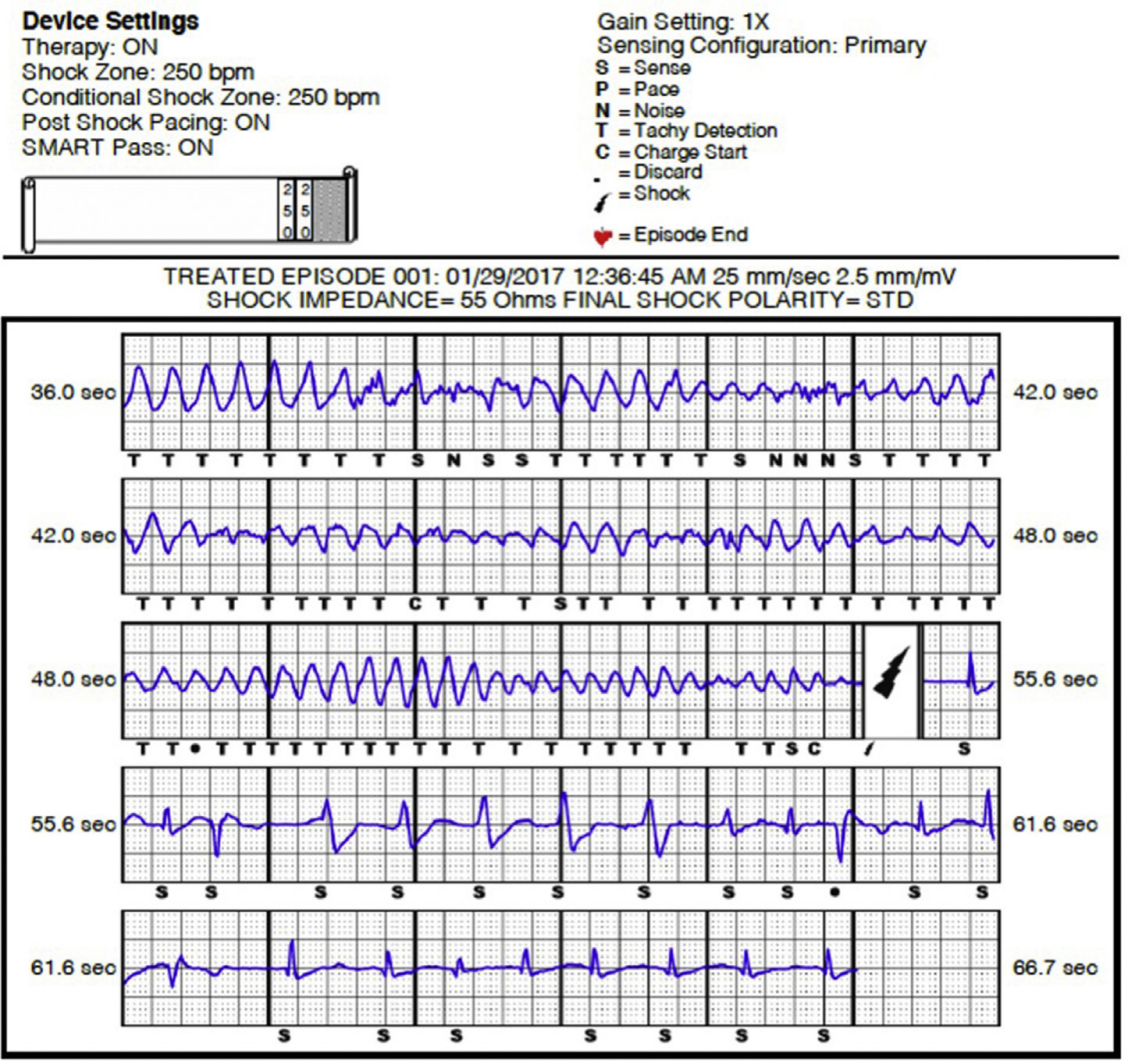
Implantable Cardioverter-Defibrillator Shock The patient had an episode of torsade de pointes at a rate >250 beats/min and received appropriate therapy for polymorphic ventricular tachycardia.

The patient delivered a healthy, full-term infant without complications. However, 7 weeks after the delivery, she experienced another syncopal episode at home. She presented to the emergency room and was noted to have a QTc of 499 ms. Interrogation of her S-ICD revealed appropriate detection and successful defibrillation of torsade de pointes at a cycle length of 198 ms (Figure 2). She subsequently underwent an elective tubal ligation procedure. She is currently working and raising her 3 children without any further reported syncopal episodes or ICD shocks.

## Conclusions

Our case describes a woman who was at exceptionally high risk for SCA, given 3 previous syncopal events associated with congenital LQTS, who subsequently became pregnant. Our case provides evidence that an S-ICD can be safely implanted during pregnancy without the risk of radiation exposure and should be considered in patients who are at high risk for SCA during the peripartum period. This case also highlights the importance of a multidisciplinary team approach when caring for pregnant cardiac patients.

## Funding Support and Author Disclosures

Dr. Gupta has received modest research grants from Medtronic; and has served as a consultant for Medtronic, Boston Scientific, and Respicardia; none of these are relevant to this paper. Dr. Myadam has reported that he has no relationships relevant to the contents of this paper to disclose.

## References

[1] Rosen M.A. (1999). Management of anesthesia for the pregnant surgical patient. Anesthesiology.

[2] Brouwer T.F., Miller M.A., Quast A.F.B.E. (2017). Implantation of subcutaneous implantable cardioverter defibrillator: an evaluation of 4 implantable techniques. Circ Arrhythm Electrophysiol.

[3] Al-Khatib S.M., Stevenson W.G., Ackerman M.J. (2018). 2017 AHA/ACC/HRS guideline for management of patients with ventricular arrhythmias and the prevention of sudden cardiac death: executive summary: a report of the American College of Cardiology/American Heart Association Task Force on Clinical Practice Guidelines and the Heart Rhythm Society. J Am Coll Cardiol.

[4] Abello M., Peinado R., Merino J.L. (2003). Cardioverter defibrillator implantation in a pregnant woman guided with transesophageal echocardiography. Pacing Clin Electrophysiol.

[5] Payne J., Lo M., Paydak H., Maskoun W. (2017). Near-zero fluoroscopy implantation of dual-chamber pacemaker in pregnancy using electroanatomic mapping. Heart Rhythm Case Rep.

[6] Viani S., Zucchelli G., Paperini L. (2016). Subcutaneous implantable defibrillator in an acromegalic pregnant woman for secondary prevention of sudden cardiac death: when (2) technologies save (2) lives. Int J Cardiol.

[7] Eldar M., Griffin J.C., Van Hare G.F. (1992). Combined use of beta-adrenergic blocking agents and long-term cardiac pacing for patients with the long QT syndrome. J Am Coll Cardiol.

[8] Knops R.E., Olde Nordkamp L.R.A., Delnoy P.H.M. (2020). Subcutaneous or transvenous defibrillator therapy. N Engl J Med.

[9] Kleemann T., Becker T., Doenges K. (2007). Annual rate of transvenous defibrillation lead defects in implantable cardioverter-defibrillators over a period of >10 years. Circulation.

[10] Wilkoff B.L., Fauchier L., Stiles M.K. (2016). 2015 HRS/EHRA/APHRS/SOLAECE expert consensus statement on optimal implantable cardioverter-defibrillator programming and testing. Heart Rhythm.

